# Three-dimensional trajectories and network analyses of group behaviour within chimney swift flocks during approaches to the roost

**DOI:** 10.1098/rspb.2016.2602

**Published:** 2017-02-22

**Authors:** Dennis J. Evangelista, Dylan D. Ray, Sathish K. Raja, Tyson L. Hedrick

**Affiliations:** 1 Biology Department, University of North Carolina at Chapel Hill, NC 27510, USA; 2 Department of Weapons and Systems Engineering, United States Naval Academy, Annapolis, MD 21402, USA

**Keywords:** chimney swift, flocking, aggregation, swarming, network, flight biomechanics

## Abstract

Chimney swifts (*Chaetura pelagica*) are highly manoeuvrable birds notable for roosting overnight in chimneys, in groups of hundreds or thousands of birds, before and during their autumn migration. At dusk, birds gather in large numbers from surrounding areas near a roost site. The whole flock then employs an orderly, but dynamic, circling approach pattern before rapidly entering a small aperture *en masse*. We recorded the three-dimensional trajectories of ≈1 800 individual birds during a 30 min period encompassing flock formation, circling, and landing, and used these trajectories to test several hypotheses relating to flock or group behaviour. Specifically, we investigated whether the swifts use local interaction rules based on topological distance (e.g. the *n* nearest neighbours, regardless of their distance) rather than physical distance (e.g. neighbours within *x* m, regardless of number) to guide interactions, whether the chimney entry zone is more or less cooperative than the surrounding flock, and whether the characteristic subgroup size is constant or varies with flock density. We found that the swift flock is structured around local rules based on physical distance, that subgroup size increases with density, and that there exist regions of the flock that are less cooperative than others, in particular the chimney entry zone.

## 1. Introduction

The movement of groups of animals, especially the coordinated behaviour of birds in flocks, has excited observers and researchers for many years leading to studies from a variety of biological and mathematical perspectives. This work has focused on identifying the implications of simple, local rules on the formation or disintegration of flocks [1,2] along with the implications of such rules for information transfer among members [3] in the presence of uncertainty [4] about what others nearby are doing. As the pairwise interactions, between any two birds in the flock, that underpin flock behaviour become more clear, interest is also shifting towards understanding the utility of flocks for purposes ranging from predator avoidance, navigation, and locomotor efficiency [5–8] and the effect of social dominance on interactions within the flock [9]. Some of the behavioural details of these underlying tasks may in turn affect how pairwise and higher-order local rules may govern flocking behaviour.

Many generalized flocking models include the presence of local, spatially based interaction rules which explain the capacity for synchronization or consensus in the absence of leaders or other means of communication [2,10,11]. In some species of flocking birds, evidence for the existence of such rules has been demonstrated [9,12]. Here we use long duration (≈30 min), high temporal resolution (30 frames s^−1^) three-dimensional tracks reconstructed from video recordings of a flock of ≈1 800 chimney swifts (*Chaetura pelagica*) circling and landing in a chimney at dusk, to probe underlying local interaction rules and variation in flock interactions and structure spatially and with time. We hypothesized that, like European starlings (*Sturnus vulgaris*) [13], but unlike many other group behaviour models [1,2], chimney swift interaction rules are based on topological distance (e.g. the *n* nearest neighbours regardless of their distance) rather than physical distance (e.g. neighbours within *x* m, regardless of number). We attempt to show this by computing conditional means of our bird interaction metric (similarity in three-dimensional heading) with respect to pairwise topological and physical distances. If the birds follow topological rules, this interaction metric should be mostly constant with respect to topological neighbour and independent of neighbour physical distance, whereas the opposite would be true if the birds use physical distance rules. While this is a simpler approach than some maximum entropy models for flocking rules proposed [14], it is less computationally expensive (an important consideration given the size of our dataset), less sensitive to local variations which we hypothesize exist here owing to the chimney target, and takes advantage of the changes in flock density that occur during the continuous 30 min recording.

While local interaction rules are hypothesized to exist in the general case, we also expect that birds are subject to spatially and temporally variable cooperative and competitive pressures, constantly balancing desired individual actions with those of the group [15]. The flock itself is, in essence, cooperative, in that the birds broadly share the same heading when at the same location and the overall circular flight pattern allows the birds to pass close to the chimney without experiencing the same degree of collision risk as would be the case if all birds converged directly on it. However, the limited opening diameter of the chimney results in a narrow navigational channel of limited capacity, suggesting that birds must compete to actually enter it before sunset and achieve a favourable position within the roost. Thus, we hypothesize the existence of subsections of the flock, especially approaching the chimney during landing, which are less cooperative than others. Alternatively, although we expect entry to the chimney to be competitive, chimney entry also appears to be the most challenging flight task within the flock, and could instead be a plausible location for highly cooperative direct leader–follower relationships, where one bird might follow another into the chimney. We test these hypotheses by spatially mapping the local similarity of heading and network metrics indicative of subgroups within the flock, to reveal regions of relatively greater or lesser cooperation, and by comparing the trajectories of birds entering the chimney at the same time with those of birds passing through a nearby control volume.

## 2. Methods and materials

### (a) Animals and video recording

We recorded freely behaving, wild chimney swifts (*C. pelagica*) in the field as they entered an overnight roost in Raleigh, NC (127 West Hargett Street, N35°; 46′; 41.3688″, W78°; 38′; 29.9340″) from a vantage point on the top of a nearby parking garage on 1 September 2014. Permission was secured to work at all private sites.

**Figure 1.**
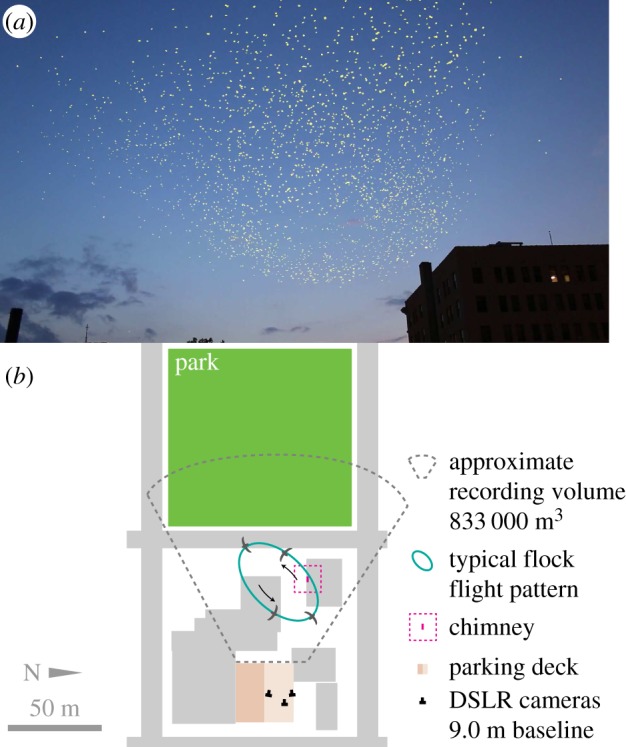
(*a*) Example video frame shows the chimney, protruding from the building in the lower right, and chimney swifts. The image was processed with background subtraction to highlight the birds in yellow. See also electronic supplementary material, movie S1. (*b*) A schematic of the field set-up shows the cameras on the parking deck, approximate imaging volume in two dimensions, the chimney, and a typical late-evening flock pattern.

Recording generally followed previously published methods [16–18]. We filmed birds from a distance of 80–150 m using three Canon EOS 6D digital SLR cameras equipped with 35 mm f/1.2 lenses placed along a 9 m transect and staggered in height. These cameras continuously recorded 1 920 ×1 080 pixel video at 29.97 frames s^−1^ through the evening. See figure 1 for an example movie frame and schematic scene layout, and electronic supplementary material, movie S1. The audio track of the recordings was used to provide a time synchronization signal [18] distributed via a set of two-way portable radios (Motorola MH230R). Weather conditions during the flock formation and entry were 26.7°C, 82% relative humidity, and wind speed less than 1.5 m s^−1^. The cameras were calibrated to allow reconstruction of three-dimensional position from the two-dimensional images. The bird trajectories were extracted using automated bird detection and track assignment routines based on [19], see the electronic supplementary material, Methods for details. Also, electronic supplementary material, movies S2 and S3 show animations of the reconstructed flock from the camera and overhead views. Chimney entries were recorded whenever a bird track terminated within 1.25 m of the centre of the chimney top. The chimney opening was rectangular, 1.2 × 1.0 m and elevated 2.6 m above the top of the building.

### (b) Individual bird and flock metrics

We computed several quantities which depended only on individual bird trajectories or on the flock considered as an unstructured group. These included the ground speed of the birds, their elevation above the chimney, the average radius of curvature of their flight path, the angular momentum of the whole flock, and the average distance to the nearest neighbour bird. Whole-flock angular momentum was computed by treating each bird as a 21.33 g point mass revolving around the flock centroid. The resulting quantity provides a combined measure of the number of birds present and the degree to which they are circling unidirectionally around the chimney. Nearest neighbour distance is the physical distance to the next closest bird; its average value quantifies flock density, while its distribution may be relevant to local behavioural rules. Pairwise distances grow *O*(*n*^2^) for each frame analysed, thus the plotted distributions (figure 3) are for samples drawn from time slices used in network computations (§2d) or from the full dataset.

### (c) Network construction and analysis

To characterize the behaviour of the flock as a set of individuals moving together we first computed the similarity in three-dimensional heading over the prior 45 video frames (i.e. 1.5 s of flight) for every possible pair of birds in each video frame subjected to network analysis. Results were stored in matrix *W*, in which *W*_*ij*_ is the similarity of heading between the *i*th and *j*th bird where 1.0 represents parallel and 0.0 anti-parallel headings. Thus, *W* as a whole describes the flock interaction network at a single instant in time, with *W*_*ij*_ specifying the edge weight between the *i*th and *j*th birds in a time-varying social network. The network defined by *W* in this manner has every bird connected to every other; thresholding was used to keep edges with greater than 95% similarity in heading for downstream computations because these would otherwise be swamped by a huge number of weak connections. We investigated the effect of distance, both physical and topological, on *W*_*ij*_ by computing its average value for a neighbour *j* at a given physical or topological distance from a focal bird *i*. We also quantified the local similarity in heading (trajectory alignment) as the summed thresholded weight, or the sum of all *W*_*ij*_ > 0.95, i.e. pairs with >95% similarity in heading, for the reasons above.

In further network-based analysis, we trimmed *W* at the highest weight (i.e. greatest similarity in heading) that left all birds connected in each time step. We then applied Suykens' synchronization-based algorithm [20] to *W* to construct a dendrogram representing hierarchical clustering of the birds in each time step, for which we computed Newman's weighted measure of modularity *Q*_w_ [21] to identify the strength of association within the flock at different group sizes. This measure, *Q*_w_, quantifies the difference in summed edge weight between the observed grouping and a random grouping of the same size; the max-modularity group size is then a measure of the group size (i.e. number of individuals) that captures the largest share of organization. For analysis of spatially binned data (see below), we extended this concept to define spatial modularity (*Q*_s_) by normalizing the max-modularity group size to the number of birds present, thereby accommodating flock regions with different bird densities. Larger values of *Q*_w_ and *Q*_s_ indicate more, smaller groups than expected for the number of birds present and smaller values indicate fewer, larger groups.

Suykens' algorithm [20] provides an estimate of branch length in addition to the dendrogram structure; this can be used to compute the probability of observing a given group size *α* at the merger of two branches, estimated within a time slice (§2d). *α* quantifies the probability of reaching a certain group size at a given level in the dendrogram, and shows whether dendrogram structure is weighted to large groups near the tips or large groups closer to the root. The dendrogram and the characteristic subgroup size *α* were used to investigate the impact of changes in flock density on flock structure. A full listing of equations to implement this analysis is provided in the electronic supplementary material, Methods.

### (d) Temporal and spatial binning

We computed the individual bird and flock metrics for the entire dataset, but selected three 750-frame and one 500-frame (duration 25 and 17 s, respectively) time slices for the computationally expensive network construction and analysis. We analysed slices in which a large number of birds were present in the recording volume, and the flock used a single elliptical approach pattern throughout, rather than during transient events such as a reversal in direction around the chimney, a split into two separate approach patterns, or merging of two patterns into one. In each of these time slices, we quantified how the average bird metrics and the properties of the network varied spatially to address our hypothesis that the flock would become more competitive (i.e. less cooperative) near the chimney roost. This was accomplished by binning the birds by their angle *θ* from the mean centre of the flock ellipse in the *x* − *y* plane relative to the chimney. We used bin sizes of *θ* = 2.5°, aggregating the entire time slice of flock activity and network metrics in each bin. For the network dendrogram analyses, we used 65 frames sampled from within each time slice owing to the even greater computational expense of this analysis. Several other computationally intensive analyses were conducted over a smaller sample of frames; details are presented with individual results.

### (e) Leader–follower pairs at landing

In order to investigate our hypothesis that the final landing flights were composed of a leader and one or more followers, we identified landing events as bird tracks that terminated within a 1.25 m radius of the chimney. We compared results for these birds to an alternate set, birds that entered into a 1.25 m radius sphere placed 6 m above the actual chimney location. From each of these sets, we identified putative leader–follower pairs as birds that either landed or passed through the control volume within 10 frames (0.3 s) of one another. We also examined the distribution of entry times at the chimney and control volume to see if chimney entries were clumped or distributed in time compared to elsewhere in the flock.

## 3. Results

### (a) Individual-based results and the overall time course of events

**Figure 2.**
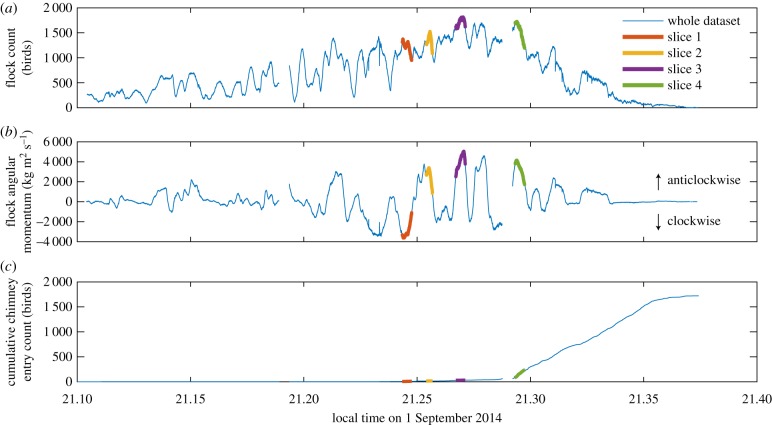
Flock count (*a*), the number of individual birds in the recording volume at a particular time, (*b*) flock angular momentum, and (*c*) the cumulative entry of birds into the chimney. Flips in the angular momentum sign denote reversals in the circling direction. The four highlighted slices were used in the network analysis and related metrics given in table 1. Gaps in the data occurred when the cameras closed one movie file and began another during recording.

Figure 2 shows results from 1 September 2014. Swifts began gathering at the roost site at approximately 21.10, ordered circling of the roost site began at 21.14, and the birds completed (or abandoned) chimney entry by 21.36, after a total of 1 720 were observed to enter the chimney. The size of the flock within the camera recording volume peaked at 1 817 birds at 21.27. The flock generally circled in a single elliptical loop with a long edge in the direction of the setting sun passing over the chimney roost. Birds on the far side of the loop from the chimney were usually approaching towards the cameras, whereas those on approach to the chimney were usually moving away from the camera (figure 1), though rotation reversed direction several times for unknown reasons. Table 1 provides the 5th, 50th, and 95th percentiles for elevation, flight speed, radius of curvature, and nearest neighbour distance at the four time slices highlighted in figure 2. Flight speeds in the flock were approximately 6 m s^−1^, substantially less than the approximately 12 m s^−1^ reported for chimney swifts engaged in foraging [22]. Median nearest neighbour distance decreased with time but remained greater than 2× wingspan throughout.

**Table 1.** Simple whole-flock quantities.

	slice 1 (*n* = 914 286)	slice 2 (*n* = 679 996)	slice 3 (*n* = 1 286 597)	slice 4 (*n* = 1 151 386)
start time (h.mm.ss)	21.24.20	21.25.24	21.26.42	21.29.19
end time (h.mm.ss)	21.24.45	21.25.40	21.27.07	21.29.44
flock direction	clockwise	anticlockwise	anticlockwise	anticlockwise
elevation above chimney (m)	4.19, 14.43, 24.71	2.99, 9.73, 16.49	2.66, 9.30, 16.78	0.20, 5.43, 11.30
ground speed (m s^−1^)	5.21, 6.88, 9.00	4.96, 6.60, 8.74	4.97, 6.56, 8.98	4.34, 6.61, 8.84
radius of curvature (m)	7.79, 21.91, 132.9	6.13, 19.49, 91.26	5.14, 17.82, 67.56	3.25, 15.32, 57.66
nearest neighbour distance (m)	0.93, 2.14, 4.99	0.76, 1.79, 4.50	0.67, 1.57, 3.62	0.51, 1.29, 3.02

Flock direction is specified as in an overhead view (figure 1*b*). Triplet results are the 5th, 50th, and 95th percentiles, compiled directly from the entire set of data points in the slice rather than from individual bird means.

### (b) Evidence for local interaction rules and global scaling

**Figure 3.**
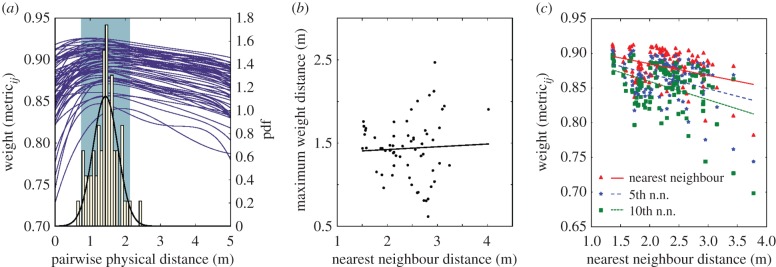
(*a*) Blue lines correspond to the left axis and are the smoothed average relationship between pairwise physical distance and heading similarity for all birds in 62 frames pulled randomly from the four time slices. Smoothing was by spline with a local standard deviation tolerance. The right axis shows the probability density scale for the histogram of maximum weight distances and its fitted normal distribution (*μ* = 1.435183, *σ* = 0.354939) 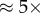 wingspan; normality was confirmed with an Anderson–Darling test (test statistic 0.313587, *p* > 0.15, null hypothesis of normality). The shaded region is the 95% CI for the mean. (*b*) The maximum weight pairwise distance did not vary with mean nearest neighbour distance (*R*^2^ = 0.002315, *p* = 0.710362). However, as shown by the trend lines in (*c*), the average weight of the *n*th neighbour decreased with increases in nearest neighbour distance, (*R* = −0.353969, −0.378956, −0.394210 for the nearest, 5th nearest, and 10th nearest neighbours, respectively; *p* = 0.000112, 3.21440 ×10^−5^, and 1.42674 ×10^−5^). As expected, average weight also declined with neighbour index. Data in (*c*) were computed from 114 samples of 100 consecutive frames across the full dataset.

Figure 3 shows how physical and topological distance in the flock affect *W*_*ij*_, the similarity in heading. In the case of physical distance, the strength of the relationship varied with time but typically decreased for distances less than 1 m and was maximal at a distance of 1.4 m (figure 3*a*); the distribution of maxima was normal and did not vary with nearest neighbour distance (figure 3*b*) or time. However, we found that the mean *W*_*ij*_ of the 1st, 5th, and 10th nearest neighbour decreased as the physical distance to that neighbour increased (figure 3*c*).

**Figure 4.**
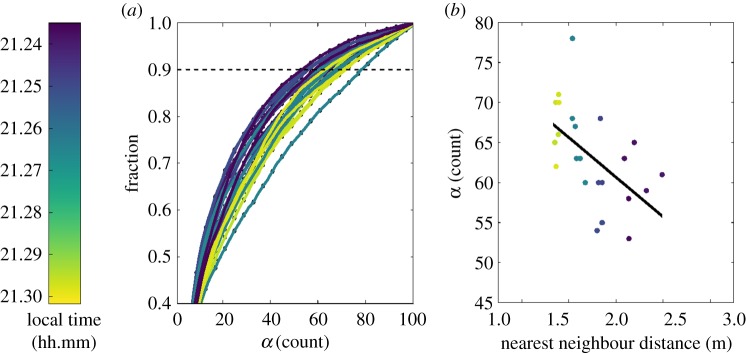
Scaling of group sizes within the flock dendrogram. Panel (*a*) shows the summed transition probabilities reaching group size *α* computed for 24 different temporally close samples of dendrograms gathered from regions of 65 consecutive frames in all four time slices. Panel (*b*) shows a linear fit to the highest integer *α* such that the summed transition probabilities are <0.9, *R* = −0.540670, *p* = 0.006375; the fit produced residuals with an estimated mean *μ* = 1.421085 × 10^−14^ and an Anderson–Darling test for normality was performed which yielded a statistic of 0.268692, 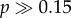 (null hypothesis of normality).

To reveal scaling of group size with flock density, we used the trimmed, weighted networks described above to obtain hierarchical information about the flock using Suykens' algorithm [20] (see electronic supplementary material, figure S4). Figure 4*a* shows the distribution of summed transition probabilities in reaching group size *α*, whereas figure 4*b* gives *α* versus the nearest neighbour distance for the 90% transect (dotted line in figure 4*a*) as a measure of the scaling between group size and nearest neighbour distance. Group size increased as the evening progressed (figure 4*a* dark on left, lighter on right) and as nearest neighbour distance decreased (figure 4*b*).

### (c) Spatial and temporal variation in flock network properties

**Figure 5.**
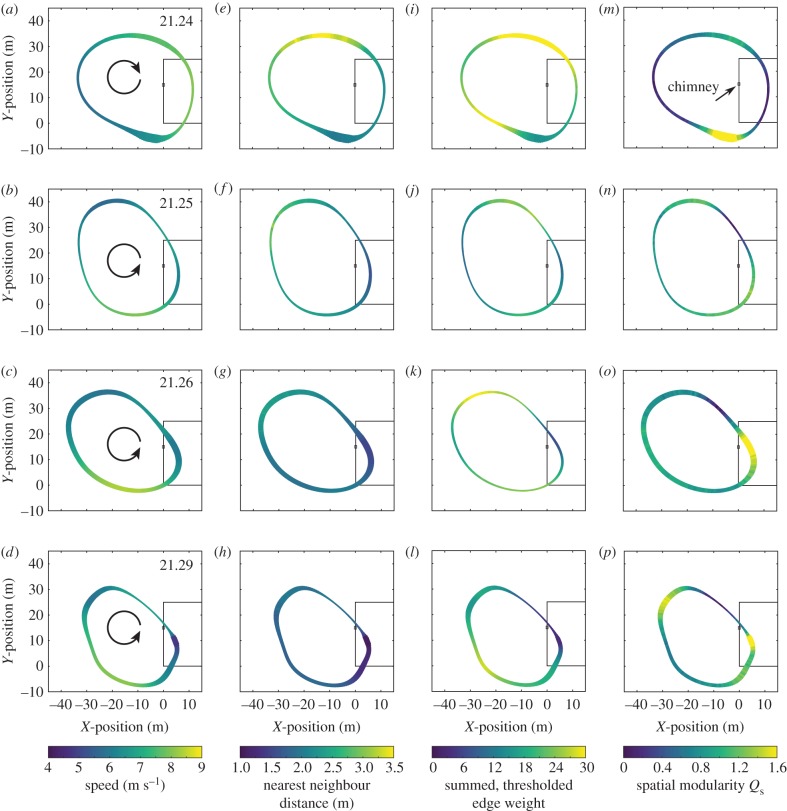
The flock properties change over time and space. Each panel shows the local average per-bird value of a flock property during a time slice, shown along the average flock path for that slice. Time slice varies by row, ranging from flock marshalling (top) to chimney entry (bottom figure 2). Columns 1–4 show, from left to right: speed, nearest neighbour distance, summed thresholded edge weight, and spatial modularity. Flock circling direction is shown in column 1. The chimney building is shown in outline on the right side of the plots and the chimney itself is marked by a small open square. The width of the ribbon indicates the number of observations in each spatial bin.

As expected, virtually all properties of the flock from speed and nearest neighbour distance to summed network weight and max-modularity group size vary spatially and temporally (figure 5). Speeds tend to be highest as the birds approach the building but then, aside from the earliest time slices, decrease once overflying it and approaching the chimney. Speed also remains largely constant through the different time slices (table 1), such that spatial variation in speed is larger than temporal variation. Some variation in speed may be due to local weather conditions; although reported as negligible, even a 1 m s^−1^ wind of consistent direction would produce a detectable 2 m s^−1^ fluctuation in ground speed as birds circle the landing site, assuming they maintain a constant airspeed. However, a comparison of clockwise and anticlockwise results (figure 5) suggests that this effect cannot explain all the observed variation.

Nearest neighbour distance tends to be greatest at points furthest from the chimney and least near the chimney, especially in the later time slices as the flock height decreases and the birds get closer to the landing site (figure 5*e*–*h*). Nearest neighbour distance for the whole flock decreases with time, and the magnitudes of the spatial and temporal differences are approximately similar. For the four time slices considered as a whole, speed and nearest neighbour distance are unrelated to one another except at the smallest distances where they are positively correlated (electronic supplementary material, figure S5).

Local similarity in heading, computed as the summed thresholded weight for edges with weights > 0.95, is typically least near the chimney (dark, thin sections at approaches to chimney, bottom right corners of panels figure 5*i*–*l*) and highest opposite it (light bands past the chimney and in the return path). This broadly coincides with patterns in flight speed and nearest neighbour distance; high thresholded edge weight is positively correlated with speed (electronic supplementary material, figure S6) and nearest neighbour distance (electronic supplementary material, figure S7). Speed and nearest neighbour distance themselves are uncorrelated in these data (electronic supplementary material, figure S5), thus effects on local similarity in heading (i.e. summed thresholded weight) are additive.

### (d) Leader–follower pairs at landing

**Figure 6.**
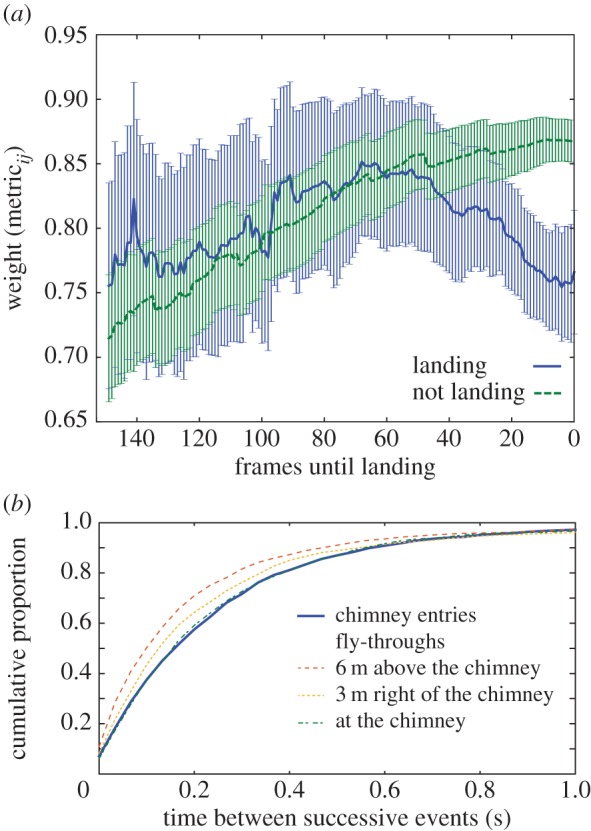
Properties of birds at landing. (*a*) Compares the network weights of pairs *W*_*ij*_ of landing and non-landing birds through time. Pairs either land or, for non-landing birds, enter a control volume 6 m above the chimney within 10 video frames of one another. Lines show the mean and 95% CIs; *n* = 65 pairs for landing and 171 for non-landing. Panel (*b*) shows the cumulative distribution of time intervals between successive landing events or birds flying through three different control regions. In this case, the volume of the control region target was adjusted to give equal number of individuals in all landing and non-landing cases. All results are from the last time slice, which contains the majority of landing events observed (figure 2).

Figure 6*a* shows the average network weight and its 95% CI for pairs of birds that either enter the chimney or pass through a control volume at nearly the same time. Non-landing pairs exhibited a monotonically increasing heading similarity as they approached the control volume while landing pairs exhibited a peak similarity approximately 70 frames before entry which then declined as they approached the chimney. The distribution of time between successive landing events was also similar to the distribution between fly-through events at several other locations near the chimney (figure 6*b*).

## 4. Discussion

We found that, contrary to our initial hypothesis, the chimney swift local interaction rules are more consistent with physical distance metrics (e.g. align to all birds between 1.5 and 3.0 m distant) rather than topological metrics (e.g. align to the closest seven birds). As discussed below, this may allow the swifts to better accommodate the fluctuations in density that occur as the birds approach the chimney roost. We also found substantial spatial variation in several flock network metrics (figure 5), including local similarity in heading (quantified as summed thresholded edge weight) and spatially localized group size and number (quantified as spatial modularity, *Q*_s_). For both these metrics, cooperation declined as the birds approached the chimney and increased immediately after a chimney flyby. We found no support for leader–follower pairs entering the chimney. Indeed, the opposite was true and birds that enter the chimney at similar times do so with significantly less well-aligned flight headings than birds that enter into (and fly through) a nearby control volume.

### (a) Groups and flock behavioural characteristics

We found that the physical distance at which two birds had, on average, maximal heading similarity did not vary with flock density as expressed by nearest neighbour distance (figure 3). This was the case even though the average nearest neighbour distance itself was both larger and smaller than the distance of maximal heading similarity at different times during the recording period (figure 3*a*,*b*, drawn from 62 frames subsampled from the four time slices analysed using network methods). Given this result, it must also be the case that the average weight of the *n*th nearest neighbour should vary with physical distance. This was verified to be true in a non-overlapping set of data that were used for the physical distance result (figure 3*c*, drawn from 114 100-frame samples from the full dataset). Thus, our analysis supports the presence of physical distance based interaction rules that are the basis of many generalized flocking models [1,2]. To the best of our knowledge, there has been no investigation as to how observable groups derived from an alignment metric change with the density of agents. However, we expect the scaling of observable groups with agent density, as we have observed, would also be consistent with these models. Chimney swifts appear to form relatively larger subgroups at high density, and smaller subgroups at low density (figure 4), i.e. the interaction rules allow for stable flock structures as bird density changes. This might be paramount in a species such as swifts where flock density varies widely in space and time owing to the presence of a focal point at the chimney and arrival of new birds at the roosting site.

Our results do not support any fixed sized topological interaction rules, and support for this hypothesis in other bird species [13] could indicate varying degrees of adaptation for flocking among bird species. The swifts as a whole are well-aligned with their nearest neighbour and higher-index neighbours. However, the degree of alignment to the *i*th neighbour varies with distance to that neighbour as expected under physical distance behavioural rules (figures 3*c* and 4*b*), so that even if swifts limit their interaction to a finite *n* neighbours, the strength of the interaction is weighted by physical distance. For this reason, topological and physical distance metrics are difficult to differentiate without large datasets of varying animal density, possibly explaining support for topological rules recently noted in studies with much smaller groups (15–86) [23].

### (b) There are regions where the flock is more/less cooperative

As shown in figure 5*i*–*l*, summed thresholded edge weight (an indication of local similarity in heading) exhibits striking spatial variation, the range of which increases with time. The lowest weight region, especially pronounced in the third and fourth time slices after birds begin landing, is just ahead of the chimney and also after it along the typical anticlockwise flock path. This is consistent with our hypothesis that the birds compete for landing trajectories; individually idiosyncratic landing trajectories will not have similar headings, producing low weights and leaving few edges above the threshold (thin dark lines in figure 5*i*–*l*) and the average weight between pairs of birds that enter the chimney at similar times is less than that of birds passing through a nearby but non-landing region at similar times (figure 6).

Summed thresholded edge weight tends to be higher when the birds are far from the chimney and when they are flying faster (thick, lighter lines in figure 5*i*–*l*). Flight speed itself might promote cooperative behaviour, quantified here as greater similarity in heading and thus greater edge weight, because speed varies more widely than nearest neighbour distance, so faster flying swifts in the flock may need to behave more consistently with respect to their neighbours to reduce the likelihood of collisions. Speed was spatially independent of nearest neighbour distance for distances above approximately 1.7 m, but average nearest neighbour distance was positively spatially correlated with summed thresholded edge weight, possibly because larger distances between birds reduce the need for avoidance manoeuvres that reduce flight heading similarity and thus edge weight.

A similar pattern of more/less cooperative spatial regions is indicated by network spatial modularity (*Q*_s_), which reveals high modularity (i.e. a tendency towards many, small groups) in two spots: directly before the chimney and directly opposite it (figure 5*m*–*p*). These regions are surrounded by low modularity regions (directly after the chimney and its opposite). Whereas the consensus towards a circular shape of the flock allows for multiple passes in the case of failure to land as well as solidifying a common approach trajectory, the consensus towards a cyclical pattern of modularity minimizes global coordination at two decision points–landing and turning back towards the landing site—while maximizing it during the transport sides of the circle, either away from or towards the chimney.

### (c) Future work

Here we examined properties of the swift landing flock when the birds were engaged in constant direction circles as part of a simple, toroidal flock. We also observed the flock reversing direction and shifting from a circular to figure-eight pattern. We do not know why these qualitative shifts in whole-flock behaviour occurred or how they are coordinated, but believe they represent one of many potentially fruitful avenues for further investigation.

## Supplementary Material

Supplemental figures

## Supplementary Material

Supplemental methods

## Data accessibility

Supporting data can be accessed from the Dryad Digital Repository: http://doi.org/10.5061/dryad.p68f8 [24]. Data are provided as the smoothed tracks and their velocities in an HDF5 file and include the following: . The Python programs used to preprocess and generate networks are available at http://bitbucket.org/devangel77b/mswarm5; codes for network analyses are at http://bitbucket.org/devangel77b/mnetwork; both are intended to be run in parallel on a cluster.
