# Supplementary material for: Three-dimensional trajectories and network analyses of group behaviour within chimney swift flocks during approaches to the roost
Source: Proc Biol Sci. 2017 Feb 22;284(1849):20162602. doi: 10.1098/rspb.2016.2602 (PMC5326531; doi:10.1098/rspb.2016.2602)
Supplement: Supplemental figures [file rspb20162602supp1.pdf]

5 Supplemental figures

Supplemental figures for Evangelista, D, Ray, D, Raja, S and Hedrick, T. 3D trajectories and network analyses of group behaviour within Chimney Swift flocks during approaches to the roost. *Proceedings of the Royal Society B*. doi:10.1098/rspb.2016.2602.

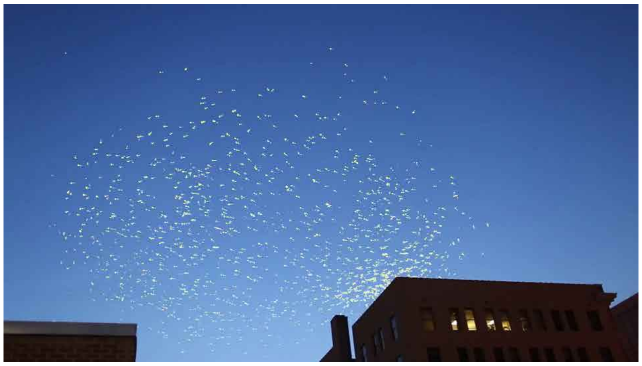

Figure S1: This thumbnail figure and the associated movie show a chimney swift landing flock with the birds highlighted in yellow via background subtraction processing. The flock is engaged in chimney entry and the highlighted bird profiles get larger as they extend their wings and slow down to attempt a landing. Note that this video was selected for its clear sky background and is not from the recording day analysed here.

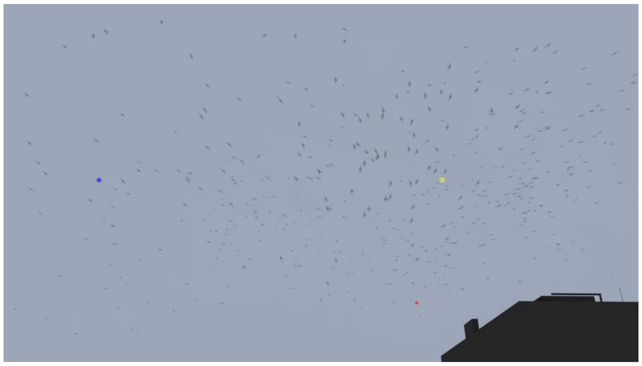

Figure S2: This thumbnail figure and the associated movie show the bird positions in a reconstructed flock from the perspective of one of the original recording cameras. The building outline was added in the 3D animation package used to produce the movie, but was not included in any analysis routines. Three individual birds are marked by colour and also shown in the overhead view movie S3.

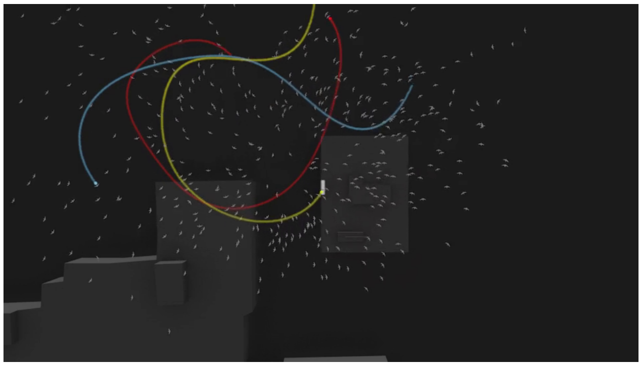

Figure S3: This thumbnail figure and the associated movie show the bird positions in a reconstructed flock from an overhead view. The building outlines were added in the 3D animation package used to produce the movie, but were not included in any analysis routines. Three individual birds and their trajectories are marked by colour; these are also shown in the camera view movie S2.

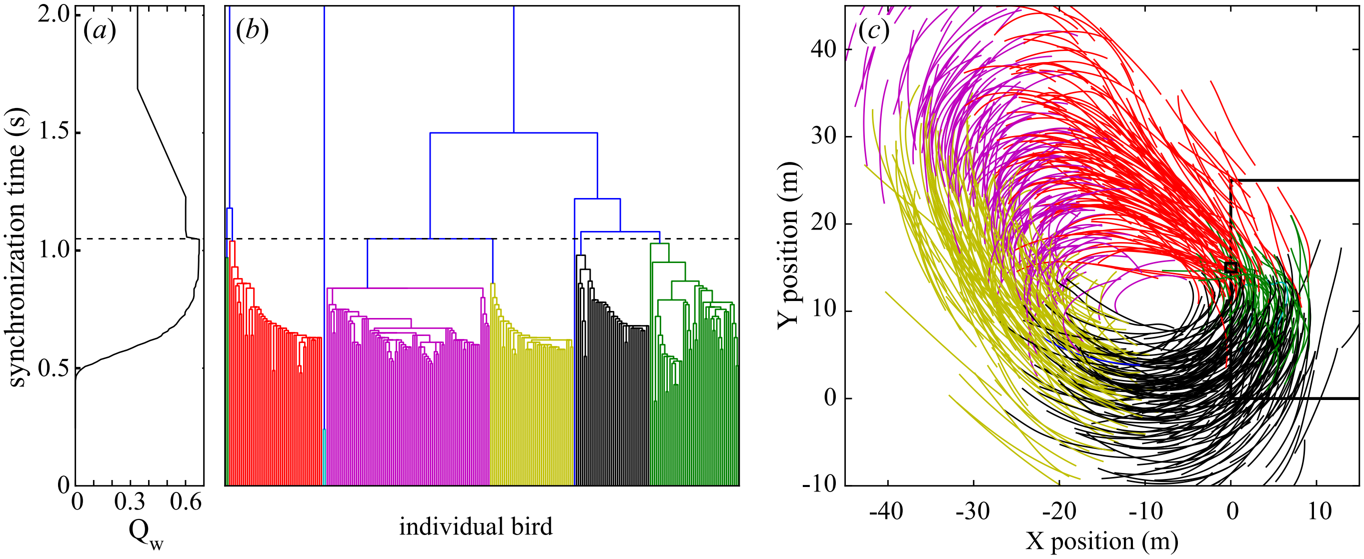

Figure S4: An example of the weighted network analysis showing in (a) the network modularity Q_w versus time to synchronisation in the community identification algorithm. The maximum Q_w from (a), shown as a dashed line, was then used to identify groups in the network community dendrogram, colour-coded in (b). The trajectories (last 2 s) of these groups are then shown in overhead flock view in (c). The flock is moving in a counter-clockwise direction, and the data are from after flock consolidation but before sustained chimney entry.

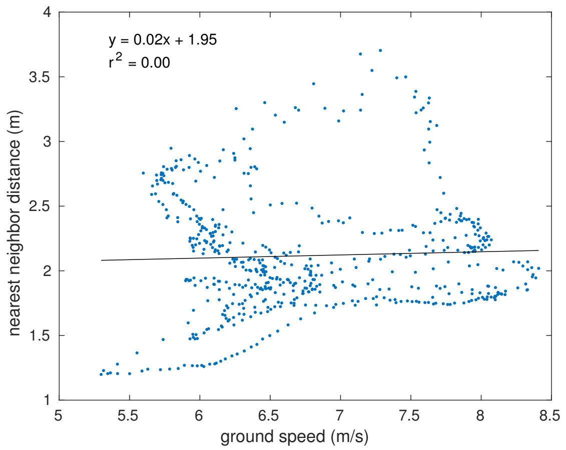

Figure S5: Speed and nearest neighbour distance are statistically unrelated to one another among the four network analysis time slices. As seen in the scatterplot, they are positively correlated for small nearest neighbour distances less than $\sim 1.7\,\mathrm{m}$, a condition only found in the fourth time slice as the birds approach the chimney. The data shown here and in the following two supplemental figures are a scatterplot of the spatially binned data shown in Fig. 5.

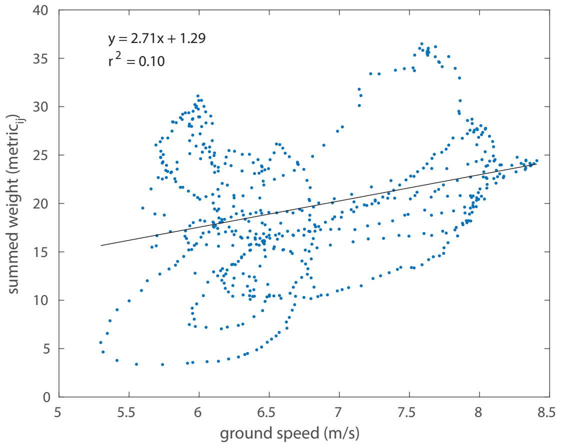

Figure S6: Speed and the summed thresholded edge weight (sum of high-weight $metric_{ij} \geq 0.95$) edges are correlated with one another among the four network analysis time slices. Among individual time slices, the effect is weak in the first two and much stronger in the second two as the flock condenses and birds begin to land.

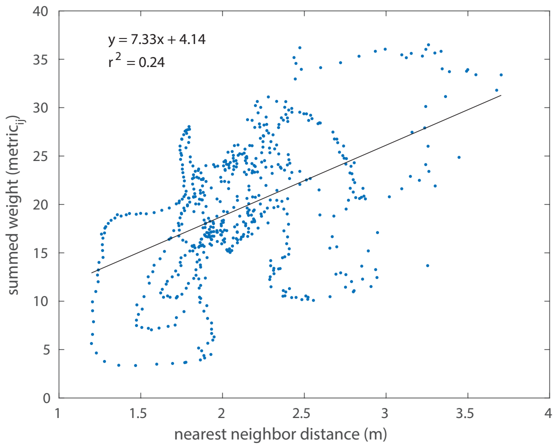

Figure S7: Nearest neighbour distance and summed thresholded edge weight (sum of high-weight ($metric_{ij} \geq 0.95$) edges are correlated with one another among the four network analysis time slices. This effect is additive with the correlation between speed and degree since speed and nearest neighbour distance are largely unrelated.
