# Supplementary material for: Three-dimensional trajectories and network analyses of group behaviour within chimney swift flocks during approaches to the roost
Source: Proc Biol Sci. 2017 Feb 22;284(1849):20162602. doi: 10.1098/rspb.2016.2602 (PMC5326531; doi:10.1098/rspb.2016.2602)
Supplement: Supplemental methods [file rspb20162602supp2.pdf]

6 Supplemental methods

Supplemental methods for Evangelista, D, Ray, D, Raja, S and Hedrick, T. 3D trajectories and network analyses of group behaviour within Chimney Swift flocks during approaches to the roost. *Proceedings of the Royal Society B.* doi:10.1098/rspb.2016.2602.

6.1 Camera calibration

Camera calibration consists of determining the intrinsic, i.e. lens and sensor characteristics and extrinsic, i.e. relative position and orientation for all three cameras. The intrinsic parameters of the camera were determined in lab prior to field recording using the OpenCV library (version 2.4) [25] applied to video of a moving 9×12 dot grid pattern. To obtain estimates of camera rotation and translation once in the field, we first digitised (i.e. recorded the pixel location of) 30 background points, primarily from buildings and permanently fixed features visible in all camera views. The background points were used to obtain an initial calibration with axes fixed to the chimney roost. The calibration was refined by manually digitising $\approx$ 1000 points from birds in the scene, adding these to the fixed background points and recalibrating. Once a sufficiently accurate calibration was obtained, automatic bird detection and matching (see below) was used to further bootstrap the calibration by adding more bird points. Test detections were made for a 750 frame slice of video and tracks greater than 50 frames in length were fed back in to refine the extrinsic calibration. The intrinsic parameters were left fixed at the values determined from the dot grid pattern. The resulting calibration provided median pixel reprojection errors of < 0.5 pixel for all cameras. The x, y, and z axes were aligned to the fixed edges of the roof of the chimney building. Scene scale was set by measurement of the length of the building. These operations used `DLTdv5` [16] to digitise points, `easyWand5` [17] to compute the calibrations, which were optimised via sparse bundle adjustment [26].

6.2 Bird detection, 3D reconstruction, track generation and smoothing

Birds were detected in each video file using background subtraction with a n-frame moving average implemented using OpenCV routines [25] in Python. The resulting background-subtracted image was thresholded and subjected to an erosion-dilation operation to remove single-pixel noise. The centroid of each individual foreground blob was then recorded and passed to the 3D reconstruction stage, implemented in MATLAB (The Mathworks, Natick, MA). The setpoint used for foreground detection was intentionally loose as false detections were eliminated during 3D reconstruction and track formation.

After generating the list of 2D blob centroids for each camera, we generated the set of 3D points by identifying all sets of three (one from each camera) 2D observations that defined a 3D point with a reprojection error (i.e. the deviation between ideal and observed 2D location) of less than 1.25 pixel. The search for sets of corresponding 2D points was sped up via epipolar gating [19], i.e. by reducing the search space dimension to linear by using camera calibration information to consider only 2D points in the second and third views close to the epipolar line determined by the 2D point under consideration in the first view. All possible sets of valid 2D observations were included, so a single observation in the first camera might be part of several 3D points, matching different observations in the second or third cameras.

Once the set of valid 3D points for each video frame was generated, we assigned these to individual bird tracks by predicting track position in 2D and 3D in frame $n+1$ using a set of Kalman filters and then using Hungarian assignment [27] to match predicted track positions to observations. The assignment operations were tiled in 30 pixel wide vertical bands with 50 % overlap to speed the assignment process. Band size was determined by examination of manually tracked trajectories, which exhibited a maximum horizontal pixel movement of $\approx 18\,\text{pixel}\,\text{frame}^{-1}$. Unmatched observations started new tracks and tracks with more than eight sequential frames with no data were stored but not considered for further matching. Tracks of less than 25 data points in total were pruned from the dataset during track formation.

The tracks were smoothed by determining the 95 % confidence interval of the camera measurement for each location in the observation volume and then using a spline to generate the smoothest (i.e. lowest magnitude first derivative) tracks that remained within the 95 % confidence intervals. Because the spatial

precision of 3D reconstruction varies substantially through the 80 m to 150 m range over which we observed the birds, tracks from more distant birds were subjected to greater smoothing than closer birds. On the whole, the 95 % confidence intervals were ≈ 10 cm in diameter, less than the ≈ 30 cm wingspan of the swifts. Velocities were determined by differentiation of the spline polynomials; smoothed tracks and their velocities were exported from MATLAB for network analysis in a high performance parallel computing environment. Sample 3D swift tracks and flock are shown in movies S2 and S3.

6.3 Network analyses

Formally, for each discrete video frame n, we define a time-varying weighted network by computing a distance metric relating all nodes (i.e. birds in the flock) to one another; the metric measured the alignment of two individuals' three-dimensional heading (unit velocity) vectors. For the pair consisting of the i-th and j-th bird at frame $[k]$:

$$e(i, j, k) = \arccos \frac{\vec{V}_i[k] \cdot \vec{V}_j[k]}{||\vec{V}_i[k]||||\vec{V}_j[k]||} \tag{1}$$

The metric is then taken as the Euclidean norm of the e values from frames $[n - m]$ to frame $[n]$; here we used m of 45 frames, or ≈ 1.5 s:

$$metric_{ij} = ||e_{ij}||_2 \tag{2}$$

and finally scaled from zero to one:

$$W_{ij} = \frac{\pi\sqrt{m} - metric_{ij}}{\pi\sqrt{m}} \tag{3}$$

This is the $i\,j$ entry of the time-dependent symmetric weight matrix W for the network of interactions. In this schema, $W_{ij} = 0$, when birds i and j are flying perfectly anti-parallel for the time window and $W_{ij} = 1$ when they are flying perfectly parallel. Note that scaling W from 0 to 1 is not strictly required, but was done to more easily compare results arrived at using variants of eqns. 1 and 2.

Our window m is over the prior frames, so our network is a lagging indicator of association and also that bird tracks of fewer than 45 prior frames cannot be part of the network even though they are part of the track dataset. Additional distance functions were tested including two dimensional (compass) heading, two dimensional velocity, and mutual shared information. We judged these less informative than the analysis presented here but all are available as part of the code repositories (see below). With up to N birds in the flock appearing in K frames the time required to exhaustively check all pairs grows as $\sim O(N^2)$. Thus, network definition was carried out in parallel over up to 512 processor nodes, using the mpi4py library [28] and custom Python routines on the UNC `killdevil` cluster. Networks were represented internally using the NetworkX Python library [29]. Intermediate computations were stored in HDF5 format [30].

We used a synchronisation based method to find dendrograms of flock structure [20]. This method creates a system of coupled oscillators whose dynamics are determined by the network of interactions between birds, i.e. W:

$$\dot{\theta}_i(t) = \omega_i + \sigma \sum_{ij} G_{ij} \sin(\theta_j(t) - \theta_i(t)) \tag{4}$$

Where θ is oscillator phase, G_{ij} is a normalised measure of interaction determined from the symmetric weight matrix W, σ is the coupling strength, and ω_i is the natural frequency of node i where the probability distribution of the frequencies can be made to be symmetric about 0. Given these conditions, birds within a group will synchronise quickly compared to those without. The dynamical system is simulated 100 times with random initial phases keeping a constant, high coupling strength $\sigma = 250$ to reduce time scales of synchronisation. The hierarchical group information and max-modularity (i.e. highest quality) groups are found by comparing the similarity in phase over all trials at each time step.

Community finding algorithms such as the one employed here make use of topological information, but by definition, the weighted network described above has no topology because each bird is connected to every other bird by some non-zero weight. To impose both a spatially consistent weighting scheme and an informative network topology, we trimmed the weighted network to the maximum weight bound that leaves

the network connected in each time step. The algorithm results in a symmetric time-dependent matrix of synchronisation times which we then processed for max-modularity groups following [20] by scrolling through the dendrogram in time steps and computing Newman's weighted measure of modularity [21]. The modularity is:

$$Q_w = \frac{1}{2W_s} \sum_{ij} \left[W_{ij} - \frac{W_i W_j}{2W_s} \right] \delta_{c_i\, c_j} \tag{5}$$

where $2W_s = \sum_{ij} W_{ij}$ is the sum of all entries in the weight matrix and the Kronecker delta $\delta_{c_i\, c_j} = 1$ when nodes i and j are within a community together, and 0 when this is not the case. This is equivalent to the fraction out of the total weight within groups minus the fraction of weight if groups were defined randomly. See Fig. S4 for an example dendrogram.

We also define a measure termed spatial modularity. It represents the degree to which a region of space occupied by the flock tends towards being unified (the presence of large groups relative to the number of individuals) or towards more granular, divisive behaviour (smaller groups). Given a series of temporally close max-modularity cuts define the function:

$$p(S) = \frac{E\big(\text{max modularity group size} \mid S\big)}{E\big(\text{number of individuals} \mid S\big)} \tag{6}$$

Where S represents a region in space where the expected values in (5) can be estimated as non-zero. By dividing by the expected number of individuals, we make these values comparable for different regions of space.

Spatial modularity, Q_S, is then defined as:

$$Q_S = -\log \left[\frac{p(S)}{\sup_S p}\right] \tag{7}$$

Here we divided by the supremum (i.e. minimum upper bound) across all spatial regions so that the log produces $Q_S = 0$ for the highest observed value of $p(S)$ and monotonically increases as $p(S)$ decreases.

The level of cooperation within temporal and spatial sections of the flock can be described using multiple network metrics. Here, we use two measures: summed thresholded weight and spatial modularity to quantify how cooperative the flock is over local and global scales, respectively. By thresholding the network edges to high (> 0.95) weights only, we restrict the information contained to only local interactions as opposed to the numerous but low weight interactions with every other bird in the flock. Spatial modularity (see above) signifies the degree of unity within a portion of the flock by measuring the tendency toward few large or many small groups as quantified by the dendrogram structure.

Dendrograms produced by the synchronisation based method allow for diagnosis of how the hierarchy of groups varies with time and flock density. We examine this by finding the probability of transitions up to a given group size α. This defines an upper triangular matrix of probabilities P such that p_{ij} is the probability of observing a group of size i becoming a group of size j. P is upper triangular due to the fact that in dendrograms groups only grow in size ("once the damage is done, it can never be repaired"). We then calculate the rate at which equation 8 approaches one with respect to group size α:

$$Pr\big(N_{i+1} \leq \alpha \mid N_{i+1} - N_i \neq 0\big) = \frac{1}{1 - \sum_{k=1} p_{kk}} \sum_{u=1}^{\alpha} \sum_{v=u+1}^{\alpha} p_{uv} \tag{8}$$

Where N_i and N_{i+1} denote the previous and next group size respectively. This is equivalent to the sum of transition probabilities given a non-zero change that end in a state (i.e. a group size) $\leq \alpha$. Of course, by definition, most transitions happen with small groups and large size transitions are less likely. However, the rate at which equation 8 approaches 1 signifies the sizes of groups which are present at lower levels of the hierarchy. If it changes slowly with α, there exists larger groups at low levels of hierarchy (i.e. close to the tips) and vice versa. Equation 8 is estimated for a small, normalised n x n region in the upper left of P so as to control for the effect of larger dendrograms; α was limited to values of 100 or less.

6.4 Methodological Limitations

Our dataset, with approximately 30 min of flock flight and more than 31 million bird positions is in many respects larger than other efforts to quantify bird flock behaviour, it is nevertheless limited in many ways. Firstly, all data are from a single flock recorded on a single evening and it is unknown how consistent the findings reported here will prove when compared to other recording days at the same location or other Chimney Swift flocks. We do plan to analyse other recording days from this flock in the future. Secondly, from a technical standpoint, our data lack persistent individual identifications longer than a few orbits, since a bird may leave the recording volume and then re-enter it, at which time it would be assigned a new identity tag. We also cannot guarantee that all birds in the recording volume have been detected and assigned to tracks. Furthermore, it is possible for a set of three different birds to be positioned in such a way as to create the appearance of a ghost bird in the 3D reconstruction step; if these occur often enough they can be joined to a create a ghost trajectory. However, the conditions that create missing or ghost bird vary with time, light levels and flock density whereas the major tests of our hypotheses are either evident throughout in the case of consistent physical distance rules or the result of temporally matched data from similar locations in the flock for competition during landing. Thus, we do not consider rare tracking defects have a substantial impact on our results.
